# Combined Treatment of Dichloroacetic Acid and Pyruvate Increased Neuronal Survival after Seizure

**DOI:** 10.3390/nu14224804

**Published:** 2022-11-13

**Authors:** Song Hee Lee, Bo Young Choi, A Ra Kho, Dae Ki Hong, Beom Seok Kang, Min Kyu Park, Si Hyun Lee, Hui Chul Choi, Hong Ki Song, Sang Won Suh

**Affiliations:** 1 Department of Physiology, College of Medicine, Hallym University, Chuncheon 24252, Korea; 2 Department of Physical Education, Hallym University, Chuncheon 24252, Korea; 3 Institute of Sports Science, Hallym University, Chuncheon 24252, Korea; 4 Neuroregeneration and Stem Cell Programs, Institute for Cell Engineering, Johns Hopkins University School of Medicine, Baltimore, MD 21205, USA; 5 Department of Neurology, Johns Hopkins University School of Medicine, Baltimore, MD 21205, USA; 6 College of Medicine, Neurology, Hallym University, Chuncheon 24252, Korea; 7 Hallym Institute of Epilepsy Research, Hallym University, Chuncheon 24252, Korea

**Keywords:** epilepsy, pilocarpine, neuron death, dichloroacetic acid (DCA), pyruvate, pyruvate dehydrogenase kinase (PDK), pyruvate dehydrogenase (PDH)

## Abstract

During seizure activity, glucose and Adenosine triphosphate (ATP) levels are significantly decreased in the brain, which is a contributing factor to seizure-induced neuronal death. Dichloroacetic acid (DCA) has been shown to prevent cell death. DCA is also known to be involved in adenosine triphosphate (ATP) production by activating pyruvate dehydrogenase (PDH), a gatekeeper of glucose oxidation, as a pyruvate dehydrogenase kinase (PDK) inhibitor. To confirm these findings, in this study, rats were given a per oral (P.O.) injection of DCA (100 mg/kg) with pyruvate (50 mg/kg) once per day for 1 week starting 2 h after the onset of seizures induced by pilocarpine administration. Neuronal death and oxidative stress were assessed 1 week after seizure to determine if the combined treatment of pyruvate and DCA increased neuronal survival and reduced oxidative damage in the hippocampus. We found that the combined treatment of pyruvate and DCA showed protective effects against seizure-associated hippocampal neuronal cell death compared to the vehicle-treated group. Treatment with combined pyruvate and DCA after seizure may have a therapeutic effect by increasing the proportion of pyruvate converted to ATP. Thus, the current research demonstrates that the combined treatment of pyruvate and DCA may have therapeutic potential in seizure-induced neuronal death.

## 1. Introduction

Epilepsy is characterized by seizures or convulsions, and it temporarily affects a person’s behavior, movement, or feelings. Moreover, by reducing the number of neurons, seizures tend to recur and usually have no immediate root cause [1]. The reason for the occurrence of epilepsy is not identified in most cases, and seizures can affect any process that the brain coordinates. The signs and symptoms of seizures may include the following: sudden body twitches or jerks, affecting the arms and legs, and, in severe cases, problems such as a loss of consciousness and abnormal sensations.

Status epilepticus is certainly a neurological emergency and could also be followed by epilepsy. This outcome was reported in 5% to 36% of children, in 22% to 41% of mixed populations of children and adults, and in 87.5% of patients with refractory status epilepticus. The production of reactive oxygen species (ROS) during refractory status epilepticus induces inflammation-related microglial cell activation and zinc release, leading to cell death [2,3]. Moreover, by reducing the number of neurons in certain regions of the hippocampus, cognitive impairment can be induced. To better understand this, we used experimental models of epilepsy that were developed to mimic epilepsy. The use of a seizure model induced by a high dose administration of pilocarpine, a muscarinic cholinergic agent, or a combination of lithium and pilocarpine in rats, has been widely adopted as an experimental research model for epilepsy in humans [4].

Pyruvate dehydrogenase (PDH) is a complex consisting of multiple copies of three enzymes: pyruvate dehydrogenase (E1), dihydrolipoamide transacetylase (E2), and dihy-drolipoamide dehydrogenase (E3) [5,6]. PDH is required to use glucose and fatty acids as energy sources and is also activated when switching to the corresponding process for ATP production. PDH regulates the influx of pyruvate into the mitochondria to initiate oxidative metabolism. Most PDH dysfunction is caused by the phosphorylation inactivation of E1 by pyruvate dehydrogenase kinase (PDK) [7,8,9]. PDK is an enzyme that inhibits the conversion of pyruvate to Acetyl-CoA in the mitochondria by inhibiting PDH activity. It regulates the oxidation of pyruvate in the mitochondria by controlling the activity of PDK, thereby regulating energy production. There are four known isozymes of PDK [10,11]: PDK1, present in the heart, skeletal muscle, and pancreatic islets; PDK2, expressed everywhere in mammalian tissues but highly expressed in the brain and particularly sensitive to metabolic signals for energy demands and fuel sources; PDK3, featuring low-level expression in most tissues except for the brain; and PDK4, normally expressed at low levels, except for in the heart, skeletal muscle, and pancreas [12,13,14].

Dichloroacetic acid (DCA) has long been studied in cancer and is a mitochondrial targeted drug that can penetrate most tissues, even after oral administration [15,16,17]. DCA is also known to improve the rate of the oxidative phosphorylation of glucose during glycolysis. The inhibition of PDK by DCA converts pyruvate to Acetyl-CoA by liberating the activity of PDH in the mitochondria. This has been shown to initiate normal oxidative phosphorylation through the TCA cycle, eventually increasing the conversion to Acetyl-CoA [18,19].

Pyruvate is an important compound in biochemistry, and it is a metabolite of glucose, produced via a process known as glycolysis [20]. Pyruvate supplies energy by being converted on the surface of the mitochondrial membrane, an intracellular organelle, to Acetyl-CoA, the main starting substrate for a series of reactions known as the citric acid cycle (or the tricarboxylic acid (TCA) cycle or the Krebs cycle). It has been found that a neuroprotective effect can be identified when pyruvate is treated in various neurological diseases and animal models, such as stroke, traumatic brain injury, and hypoglycemia [21,22,23].

In this study, based on the evidence that DCA and pyruvate have distinct neuroprotective effects in various neurological settings, DCA and pyruvate were co-administered to increase the conversion of Acetyl-CoA from pyruvic acid and increase glucose utilization. To test the hypothesis that seizure-induced neuronal death may be attenuated by increasing energy production. In this study, we hypothesized that co-administering a low dose of DCA (100 mg/kg) and a low dose of pyruvate (50 mg/kg) could be an ideal therapeutic approach to prevent neuronal cell death induced by seizures. 

## 2. Materials and Methods

### 2.1. Ethics Statement

This study was approved and carried out in accordance with the Laboratory Animal Guide published by the National Institute of Health (NIH), Chuncheon Hallym University Laboratory Animal Research Management and Utilization Committee Rules (Protocol # Hallym R1 (2018-17)). We ultimately sacrificed the animals with isoflurane anesthesia and tried to minimize their pain.

### 2.2. Experimental Animals

Experiments were conducted using 8-week-old Sprague Dawley male rats (250–350 g, Daehan Biolink (DBL) Co., Negative, Chungbuk, Eumsung Korea). Animals were kept at a constant humidity (55 ± 5%) and room temperature (22 ± 2 °C), and the room’s lights were set to automatically turn on at 12 h intervals. The guidelines for this study were designed based on Animal Research: Reporting in Vivo Experiments (ARRIVE).

### 2.3. Seizure Induction

**Figure 1:**
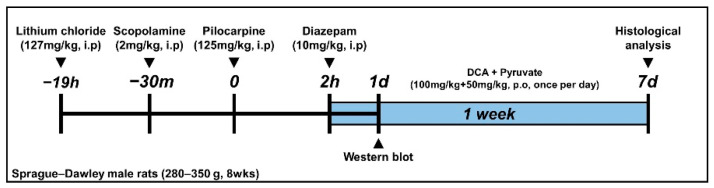
Schematic of timeline showing the pilocarpine-induced seizure and key experiments performed in this study. Seizure was induced by pilocarpine injection. Dichloroacetic acid (DCA) and pyruvate were orally administered once per day for all experimental periods.

Pilocarpine (25 mg/kg. i.p.) was injected to activate the muscarinic receptor in order to induce a seizure. Lithium chloride (LiCl, 127 mg/kg, i.p.) was injected 19 h prior to the administration of pilocarpine in order to boost the action of pilocarpine. Scopolamine (2 mg/kg, i.p.) was injected 30 min prior to the administration of pilocarpine, thereby minimizing muscarinic cholinergic side effects [24,25,26]. One rat was housed per cage, and seizure behavior was confirmed by monitoring the Racine stage [27,28]. There are 5 Racine stages ((1) mouth and facial movement, (2) head nodding, (3) forelimb clonus, (4) rearing with forelimb clonus, and (5) rearing and falling with forelimb clonus). During falling, the last Racine stage, a seizure was induced. The seizure occurred within 30 min to 1 h after pilocarpine administration. Two hours after the onset of the seizure, diazepam (10 mg/kg, i.p.) was injected to suppress it (Figure 1).

### 2.4. Dichloroacetic Acid (DCA) and Pyruvate Injection

To confirm the effect of the dichloroacetic acid (DCA) and pyruvate co-treatment, each group was divided into 8 subgroups (the sham group comprised vehicle, DCA, pyruvate, and combined DCA and pyruvate groups, and the seizure group comprised DCA, pyruvate, and combined DCA and pyruvate groups). DCA (100 mg/kg, p.o.) and pyruvate (50 mg/kg, p.o.) were administered orally once a day for a week [29]. In the vehicle group, 0.9% physiological saline was injected for one week following the same schedule (Figure 1). *n* = 5 for each sham group. *n* = 8 for each seizure group.

### 2.5. Brain Sample Preparation

After the administration of dichloroacetic acid (DCA) and pyruvate for 1 week, the animals were sacrificed. Injections of urethane (1.5 g/kg i.p.) were used to anesthetize the rats. Blood was removed from the anesthetized rats’ brains by perfusing 500–600 mL of 0.9% physiological saline into their hearts. For tissue fixation, 4% paraformaldehyde was perfused, and then the brain was harvested. Harvested brains were fixed in the same 4% paraformaldehyde for 1 h. After fixation, each brain was stored in 30% sucrose solution for cryoprotection. The brains stored in the sucrose solution sank to the floor after 2–3 days, and the whole brain was frozen using a cryostat microtome (CM1850; Leica, Wetzlar, Germany) and sliced into coronal sections with thicknesses of 30 μm.

### 2.6. Detection of PDH and PDK2

Staining was performed by attaching primary antibodies to determine pyruvate dehydrogenase (PDH) and pyruvate dehydrogenase kinase 2 (PDK2) levels in the hippocampal area of the brain. Immunotissue chemistry was performed using primary single-clone rabbit antibodies (PDH 1:100, ab168379, PDK2:100, ab68164, Abcam, Cambridge, British Cambridge, UK). After incubation for 15 h overnight, the tissue was washed with phosphate-buffered saline (PBS) for 10 min × 3 times each, and secondary antibody (PDH: Alexa Fluor 594-IgG, PDK2: Alexa Fluor 488-IgG, conjugated donkey anti-rabbit, respectively, 1:250, Molecular Probes, Invitrogen, Carlsbad, CA, USA) staining was performed at room temperature (RT) for 2 h.

For microscopic observations, the brain tissues were plated on a gelatin-coated slide, and there were 6 sections per slide. To confirm the fluorescence intensities of the PDH and PDK2 immunoreactivities, the sections were imaged using a confocal microscope (LSM 710; Carl Zeiss, Oberkochen, Germany) with excitation and emission wavelengths of 480 and 525 nm, respectively. Using ImageJ (version 1.47c; NIH, Bethesda, MD, USA), the following steps were performed on the acquired data: in each brain tissue sample, PDK2- and PDH-detected cells were selected (5 cells) and then measured using an optional analysis in the menu (Magnification = 40× and 80×). To measure the cells’ PDK2 and PDH intensities, the menu option Analyze -Measure was selected.

### 2.7. Detection of Oxidative Injury

Using a cryostat microtome (CM1850; Leica, Wetzlar, Germany) to confirm the lipid peroxidation product (that is, oxidative injury), 4-hydroxy-2-nonenal (4HNE) staining was performed on the brain tissues sectioned into coronal sections of 30 mm thicknesses [30,31,32,33,34]. The brain samples were incubated overnight at 4 °C with 4-hydroxy-nonenal (4HNE) antibody (diluted 1:500, Alpha Diagnostic Intl. Inc., San Antonio, TX, USA). The brain sections treated with the primary antibody were washed 3 times with 0.01 M PBS for 10 min and then diluted 1:250 with a secondary antibody solution (Alexa Fluor 594 anti-mouse IgG, Invitrogen, Grand Island., NY, USA) at RT for 2 h. The brain tissues were plated on a gelatin-coated slide, and there were 6 sections per slide. To confirm the fluorescence intensity of the 4HNE immunoreactivity, ImageJ (NIH, Bethesda, Rockville, MD, USA) software was used. The sequence of use was as follows: Image load, Adjust, Color threshold (brightness and saturation with same values for all samples), Change the image type 8 bit, and Measure the intensity).

### 2.8. Detection of Microglia and Astrocyte Activation

To indirectly measure post-damage inflammation, adaptor molecule 1 (Iba1) and glial fibrillary acidic protein (GFAP) staining was performed to confirm the activation of the microglial and astrocyte cells, known as indicators of an inflammatory response. The tissue was stained with the primary antibody as a mixture of monoclonal antibodies with rat Iba1 and rabbit antibodies with rat GFAP (primary Iba1diluted at 1:500 and GFAP diluted at 1:1000, Abcam, Cambridge, British Cambridge, UK). The tissue was incubated overnight in a 0.01 M PBS 4 °C incubator containing 0.3% TritonX-100, and then the tissue was washed 3 times for 10 min. The samples were immersed in the secondary antibodies Alexa Fluor 488-IgG and Alexa Fluor 594-IgG (anti-goat and rabbit 1:250 dilution; Molecular Probes, Invitrogen) for 2 h at RT. Astrocytes and microglia were measured using ImageJ (NIH, Bethesda, Rockville, MD, USA) software. For astrocytes and microglia, after photographing the same areas (scale = 20×) of both hippocampal cornu ammonis 1 (CA1) regions, five brain sections on the slide were taken, and the function of the microglia cell intensity was measured (the microglial and astrocyte fluorescence intensity signals using ImageJ were determined as follows: Image load, Adjust, Color threshold (brightness and saturation with same values for all samples), Change the image type 8 bit, and Measure the intensity) [35].

### 2.9. Detection of Neuronal Nuclei

The number of surviving neurons present was observed to determine whether nerve cell extinction from seizures could be reduced by processing dichloroacetic acid (DCA) and pyruvate. To quantify surviving neurons, we chose five brain sections from the animals used. We performed neuronal nuclei (NeuN) staining using the primary antibody anti-mouse-NeuN (1:500 dilution, Millipore, Billerica, MA, USA). The primary antibody was incubated overnight at 4 °C and then washed with PBS for 3 min; then, it was incubated at RT for 2 h in an anti-mouse IgG secondary antibody solution (1:250 dilution, Vector Labororoid, Burlingame, CA, USA). After attaching secondary antibodies to the tissue, brain samples were treated in an RT shaker for two hours with ABC complex solutions (Vector, Burlingame, CA, USA). The sample was then washed 3 times for 10 min, and 0.01 M PBS buffer (0.06% 3,3′-diaminobenzidine, DAB ager, Sigma-Aldrich Co., St. Louis, MO, USA and 30% H_2_O_2_) was added to activate the immune response. Brain tissue sections were stained for 1 min 30 s in the solution. Stained tissues were placed on the coated slide and allowed to dry. The dried slides were mounted with a Canadian balsam (Junsei Chemical, Chuo-ku, Tokyo, Japan) solution. The number of surviving neurons in a certain area (magnification = 10×) of the coronal section was counted, and the number of surviving neurons was measured using a statistical analysis. The five brain sections used were the Subiculum (900 × 1200 mm), Cornu ammonis 1 (CA1) (900 × 400 mm), Cornu ammonis 3 (CA3) (900 × 1200 mm), subiculum regions (900 × 1200 μm) from the brain hippocampal area in both hemispheres.

### 2.10. Fluoro-Jade B (FJB) Staining

Fluoro-Jade B (FJB) staining was performed to reveal degenerating neurons in the brain sections obtained 1 week after seizure. Coronary brain slices were collected using cryo-sectioned with a thickness of 30 μm. Brain sections were placed on gelatin-coated slides, was performed by immersion in 0.001% FJB solution (Histo-Chem Inc., Jefferson, AR, USA). FJB (+) cells were counted in both hemispheric hippocampus CA1, CA3 and subiculum regions. The counted number of FJB (+) cells in each region was used for statistical analysis.

### 2.11. Western Blot

A Western blotting analysis was performed to determine the protein levels of determine pyruvate dehydrogenase kinase 2 (PDK2) and pyruvate dehydrogenase (PDH) in the vehicle and dichloroacetic acid (DCA) and pyruvate co-treatment groups. Brain perfusion was performed with only saline, and then the brains were harvested. The harvested brains were dissected to the hippocampus and then homogenized in a RIPA buffer. Homogenized tissues were incubated on ice for 30 min and then centrifuged at 4 °C for 20 min at 14,000 rpm. For centrifuged samples, only the supernatant was used. The harvested supernatant was stored at −80 °C in a cryogenic freezer until use. The protein quantification of the sample was performed using a Bradford protein analysis. The quantified hippocampal protein was diluted in an SDS electrophoresis sample buffer, separated on an 8% SDS-polyacrylamide gel, and then transferred to a polyvinylidene difluoride (PVDF) membrane. Incubation in 5% skim milk was carried out for 1 h to block non-specific staining. The membrane was incubated overnight at 4 °C with monoclonal rabbit antibody against rat PDH (1:3000, ab168379 dilution, Abcam, Cambridge, UK), monoclonal rabbit a single antibody against rat PDK2 (1:2000, ab68164 dilution, Abcam, Cambridge, UK) and monoclonal rabbit antibody against rat *p*-PDH (1:2000, ab177461 dilution, Abcam, Cambridge, UK). After the primary antibody culture, the membrane was washed 3 times for 10 min in TBST (Cat. 190-6435, Bio-Rad, Hercules, CA, USA). Using a secondary antibody membrane, the membranes were incubated for 1 h at RT with anti-rabbit IgG (dilution 1: 5000; Ab frontier). This culture was created to react with a chemiluminescence imaging system device (Amersham imager 680 machines, GE Healthcare, Little Chalfont, Buckinghamshire, UK) using an enhanced chemiluminescence (ECL) solution (Cat. P90720, Millipore) prior to observation in order to determine the protein concentration.

### 2.12. Tape Removal Test (TRT)

The tape removal test (TRT) tested whether the sensory cognitive dysfunction caused by seizure was recovered when the dichloroacetic acid (DCA) and pyruvate co-treatment was administered. The experiment was evaluated by dividing it into the following steps: One sheet of adhesive tape (1 × 1 cm) was attached to the palm of one forefoot of the rats, and the behavioral results for the removal time were observed in the experimental box of the experimental animals (maximum time: 120 s) [36]. The time was noted when the rats recognized the adhesive tape on their forefoot and removed the adhesive tape with their forepaw or their mouths. TRTs were performed 5 times, and if the tape could not be removed, the removal time was considered the maximum. 

### 2.13. ATP Measurements

ATP levels were measured using ATP colorimetric/fluorometric assay (ab83355, abcam, Cambridge, MA, USA), following manufacturer’s protocols. ATP levels were quantified in the extracted samples using a colorimetric microplate reader at absorbance 570 nm (OD570).

### 2.14. Data Analysis

Nonparametric tests and blind tests were used to determine the statistical significance between experimental groups, and all data are expressed as mean ± Standard Error of the Mean (S.E.M.). Statistical significance was measured between experimental groups using an analysis of variance (ANOVA) according to a Bonferroni post hoc test. The *p*-value was considered statistically significant when it was less than 0.05.

## 3. Results

### 3.1. DCA and Pyruvate Co-Treatment Reduces Excessive PDK2 Levels after Seizures

**Figure 2:**
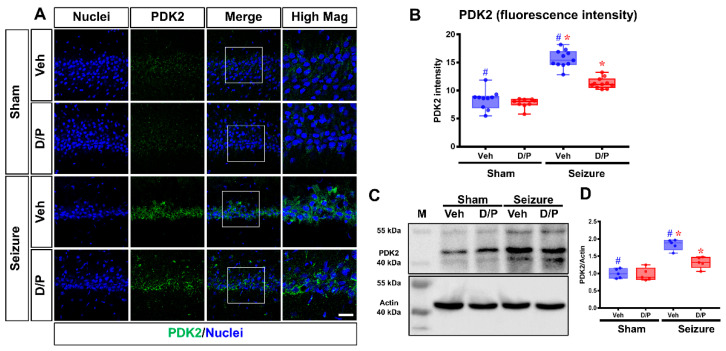
Effects of dichloroacetic acid (DCA) and pyruvate co-treatment on seizure-induced pyruvate dehydrogenase kinase 2 (PDK2) level. The effect of DCA and pyruvate co-treatment on PDK2 levels after seizure is shown in the fluorescence images. (**A**) Difference in the PDK2 intensity in the vehicle and DCA and pyruvate co-treatment groups in CA1 of the hippocampus after seizure. Scale bar = 10 μm. (**B**) The graph shows the quantification of PDK2 intensity between the groups in CA1 after seizure. Reduced the level of PDK2 in cornu ammonis 1 (CA1) area (Kruskal–Wallis test followed by a Bonferroni post hoc test: chi square = 32.664, *df* = 3, *p* = 0.018) (**C**,**D**) Neurons in a normal physiological state maintain a basal level of PDK2, while a significant increase in PDK2 is induced after seizure. However, DCA and pyruvate co-treatment reduces the excessive level of PDK2 y axis: PDK2/Actin level relative sham vehicle (Kruskal–Wallis test followed by a Bonferroni pos-hoc test: chi square = 8.457, *df* = 3, *p* = 0.037). M: marker. Data are the mean ± Standard Error of the Mean (S.E.M.).; *n* = 3 from each group. ^#,^* Significantly different from the vehicle-treated group; * *p* < 0.05.

Pyruvate dehydrogenase kinase 2 (PDK2), as a major regulator of glucose metabolism, inhibits PDH by phosphorylating it when damage occurs. In this study, it was hypothesized that increasing PDK would phosphorylates PDH and inhibits it, thereby inhibiting the process of converting pyruvate to Acetyl-CoA and reducing ATP production, resulting in neuronal cell death. Here, when PDK2 expression was evaluated by immunostaining and a Western blot (WB) analysis, it was confirmed that the level of PDK2 significantly increased by 83.1% in the seizure-induced group compared to that in the sham–vehicle group. However, in the seizure group treated with dichloroacetic acid (DCA) and pyruvate co-treatment, an inhibitor of PDK2, the intensity of PDK2 reduced by 27% compared to that in the seizure–vehicle group (Figure 2).

### 3.2. DCA and Pyruvate Co-Treatment Increased PDH Levels after Seizure

**Figure 3:**
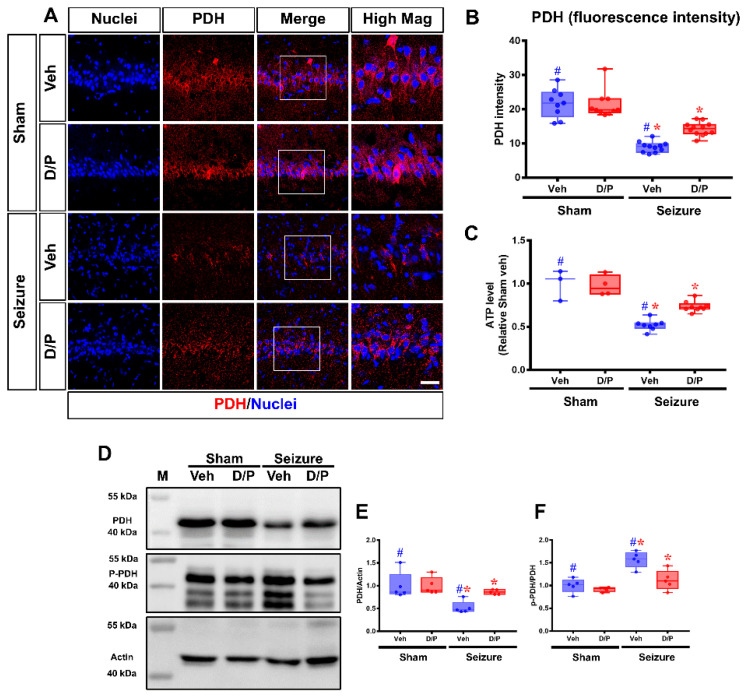
Effects of dichloroacetic acid (DCA) and pyruvate co-treatment on seizure-induced pyruvate dehydrogenase (PDH) decrease. The effect of DCA and pyruvate co-treatment on PDH levels after seizure is shown in the fluorescence images. (**A**) The fluorescence images show differences in PDH signals in CA1 between the vehicle and DCA and pyruvate co-treatment groups after seizure. Scale bar = 10 μm. (**B**) The graph shows the quantification of PDH intensity between groups after seizure. y axis: PDH intensity in CA1 area (Kruskal–Wallis test followed by a Bonferroni post hoc test: chi square = 34.587, *df* = 3, *p* = 0.025). (**C**) In the steady-state sham group, an appropriate amount of ATP level was maintained, whereas in the seizure group, the in adenosine triphosphate (ATP) level was significantly decreased. y axis: ATP level relative sham vehicle (**D**–**F**) In the steady-state sham group, an appropriate amount of active PDH was maintained, whereas in the seizure group, the level of PDH significantly decreased. However, DCA and pyruvate co-treatment increased PDH level (**E**) y axis: PDH/Actin level relative sham vehicle (Kruskal–Wallis test followed by a Bonferroni post hoc test: chi square = 6.458, *df* = 3, *p* = 0.05). P-PDHα1-Ser293 (P-PDH) was expressed in the hippocampus. P-PDH level increased in the seizure group. However, the level of P-PDH decreased in the DCA and pyruvate co-treatment group (**F**) y axis: *p*-PDH/PDH level relative sham vehicle (Kruskal–Wallis test followed by a Bonferroni post hoc test: chi square = 9.095, *df* = 3, *p* = 0.028). M: marker. Data are the mean ± Standard Error of the Mean (S.E.M.).; *n* = 4 from each group. ^#^ Significantly different from the sham vehicle and seizure vehicle-treated group, * Significantly different from the vehicle-treated group; * *p* < 0.05.

Determine pyruvate dehydrogenase (PDH) is an enzyme that synthesizes adenosine triphosphate (ATP) by converting pyruvate into Acetyl-CoA. PDH immunostaining was used to confirm whether the reduced PDH level due to increased PDK after a seizure can be increased by co-treatment with dichloroacetic acid (DCA) and pyruvate. The PDH level was confirmed in the sham group, and the PDH levels decreased as the level of PDK increased in the seizure-induced group. It was confirmed that the level of PDH significantly decreased in the seizure-induced group by 62.1% compared to that in the sham–vehicle group. However, in the seizure group treated with DCA and pyruvate co-treatment, the level of PDH increased by 42.1% compared to that in the seizure–vehicle group. In addition, we investigated changes in P-PDHα1-Ser293 (P-PDH) following seizures. P-PDH significantly increased by 62.3% in the seizure-induced group compared to that in the sham–vehicle group. However, in the seizure group treated with DCA and pyruvate co-treatment, the level of P-PDH reduced by 30.8% compared to that in the seizure–vehicle group. We found that ATP levels significantly decreased after seizure-induced group by 42.5% compared to that in the sham-vehicle group. DCA and pyruvate co-treatment increased ATP levels, even after seizure (Figure 3).

### 3.3. DCA and Pyruvate Co-Treatment Decreases Oxidative Stress after Pilocarpine-Induced Seizure

**Figure 4:**
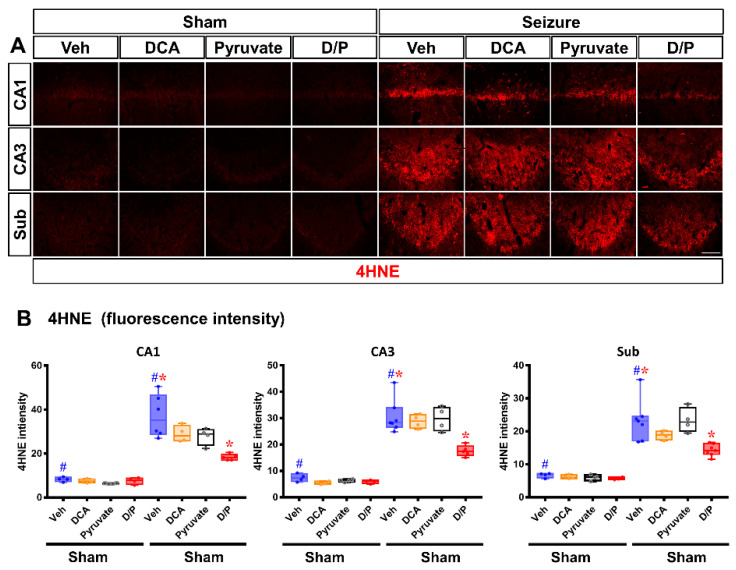
Dichloroacetic acid (DCA) and pyruvate co-treatment reduces oxidative damage after seizure. Oxidative damage of the hippocampus 7 days after seizure induction was evaluated by 4-hydroxy-nonenal (4HNE) staining in the cornu ammonis 1 (CA1), CA3 and subiculum regions. (**A**) The sham group showed minimal 4HNE immune response signals in the hippocampal region. In the seizure group, 4HNE intensity was significantly increased. DCA and pyruvate co-treatment decreased the immunoreactive fluorescence signal of 4HNE in the hippocampus after seizure. Scale bar = 100 μm. (**B**) The 4HNE fluorescence of the hippocampus is plotted as a bar graph. y axis: 4HNE intensity in CA1, CA3 and subiculum (Sub) in hippocampal area (Kruskal–Wallis test followed by a Bonferroni post hoc test: CA1 chi square = 25.275, *df* = 7, *p* = 0.01; CA3 chi square = 25.472, *df* = 7, *p* = 0.01; Sub chi square = 27.82, *df* = 7, *p* = 0.012) blue: vehicle, yellow: dichloroacetic acid (DCA), black: pyruvate, red: DCA and Pyruvate co-treatment. Data are the mean ± Standard Error of the Mean (S.E.M.). *n* = 4 for each sham group. *n* = 5–7 for each seizure group. ^#^ Significantly different from the sham vehicle and seizure vehicle-treated group,* Significantly different from the vehicle-treated group; * *p* < 0.05.

After a seizure, the reactive oxygen species (ROS), induced by the excessive activity of NADPH oxidase, cause oxidative stress [30]. Here, after seizure, 4-hydroxy-nonenal (4HNE, an indicator of lipid peroxidation) staining was performed to confirm whether oxidative stress, one of the main causes of neuronal cell death, increased [37]. There was no difference in the 4HNE fluorescence signals among the sham–vehicle and treatment groups. However, after inducing the seizure, the 4HNE fluorescence of the saline-treated group increased significantly in each hippocampal region. The dichloroacetic acid (DCA) and pyruvate co-treatment groups showed a decrease in 4HNE intensity after seizure compared to the saline-treated vehicle group (Figure 4A,B compared to the seizure–vehicle group, the seizure–DCA and pyruvate combination treatment group exhibited reduced oxidative stress in CA1 by 50.7%, in CA3 by 42.6% and in Sub by 38.6%).

### 3.4. DCA and Pyruvate Co-Treatment Prevents Inflammatory Reaction after Pilocarpine-Induced Seizure

**Figure 5:**
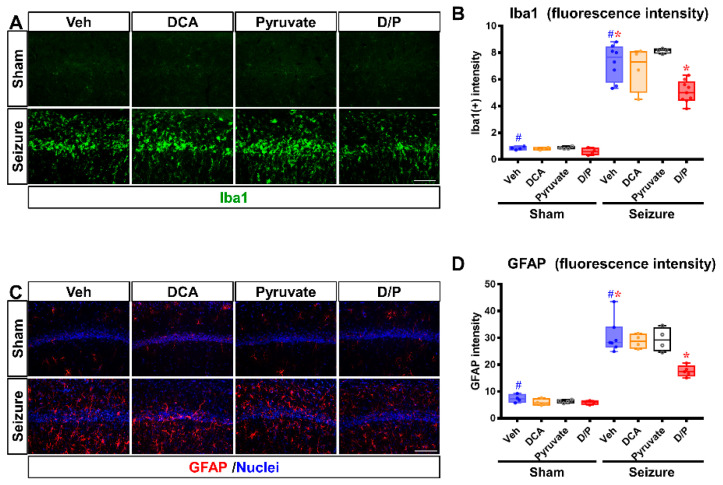
Dichloroacetic acid (DCA) and pyruvate co-treatment reduces seizure-induced activation of microglia and astrocytes. The activation of microglia and astrocytes induced after seizure was observed through adaptor molecule 1 (Iba1) and glial fibrillary acidic protein (GFAP) staining. (**A**) Iba1 shows fluorescence images of the expressed microglia in the hippocampal CA1 region. The sham group showed little microglial expression. The seizure-induced group showed the activation of microglia. However, after seizure, it was confirmed that the activation of the microglia was significantly reduced in the DCA and pyruvate co-treatment group compared to the vehicle-treated group. Scale bar = 100 μm. (**B**) Graph of the grade of Iba1 intensity in hippocampus CA1 region (Kruskal–Wallis test followed by a Bonferroni post hoc test: chi square = 28.149, *df* = 3, *p* = 0.022) blue: vehicle, yellow: dichloroacetic acid (DCA), black: pyruvate, red: DCA and Pyruvate co-treatment. (**C**) GFAP astrocyte fluorescence microscopic image of hippocampal CA1; it was confirmed that the activation of astrocytes was significantly reduced in the DCA and pyruvate co-treatment group compared to the vehicle-treated group after seizure, as in Iba1. Scale bar = 100 μm. (**D**) The graph shows quantification of activated GFAP based on the GFAP fluorescence signal. y axis: GFAP intensity (Kruskal–Wallis test followed by a Bonferroni post hoc test: chi square = 19.872, *df* = 3, *p* = 0.006). Data are the mean ± Standard Error of the Mean (S.E.M.). *n* = 4 for each sham group. *n* = 5–7 for each seizure group. ^#^ Significantly different from the sham vehicle and seizure vehicle-treated group, * Significantly different from the vehicle-treated group; * *p* < 0.05.

The sustained activation of microglia/macrophages and astrocytes can exacerbate neuroinflammation and contribute to neurodegeneration [38], were confirmed with adaptor molecule 1 (Iba1) and glial fibrillary acidic protein (GFAP) staining. Activated astrocytes can be harmful because they produce NO, which can cause neurotoxicity [39,40,41]. When the excessive activation of microglia and astrocytes persists, the prolonged production of inflammatory mediators by the microglia can induce chronic inflammation and may be associated with further tissue damage. As shown in Figure 5A,B there were no differences in the signals of the activation intensity of the microglia identified by Iba1 in the sham groups. After seizure, the intensity of the microglial fluorescence signal increased significantly. However, the dichloroacetic acid (DCA) and pyruvate co-treatment decreased microglial/macrophage expression (compared to the seizure–vehicle group, the seizure–DCA and pyruvate combination treatment group exhibited reduced Iba1 intensity by 30.3%). Figure 5C,D show the intensity of the astrocyte activity analysis through the fluorescence signal of GFAP in the CA1 region of the hippocampus. Compared to the sham group, the astrocytes of the seizure–vehicle group showed a distinct hypertrophy process, and the DCA and pyruvate co-treatment significantly reduced the activation of astrocytes after seizure (compared to the seizure–vehicle group, the GFAP intensity of the seizure–DCA and pyruvate combination treatment group reduced by 42.3%).

### 3.5. DCA and Pyruvate Co-Treatment Increases Surviving Neurons after Pilocarpine-Induced Seizure

**Figure 6:**
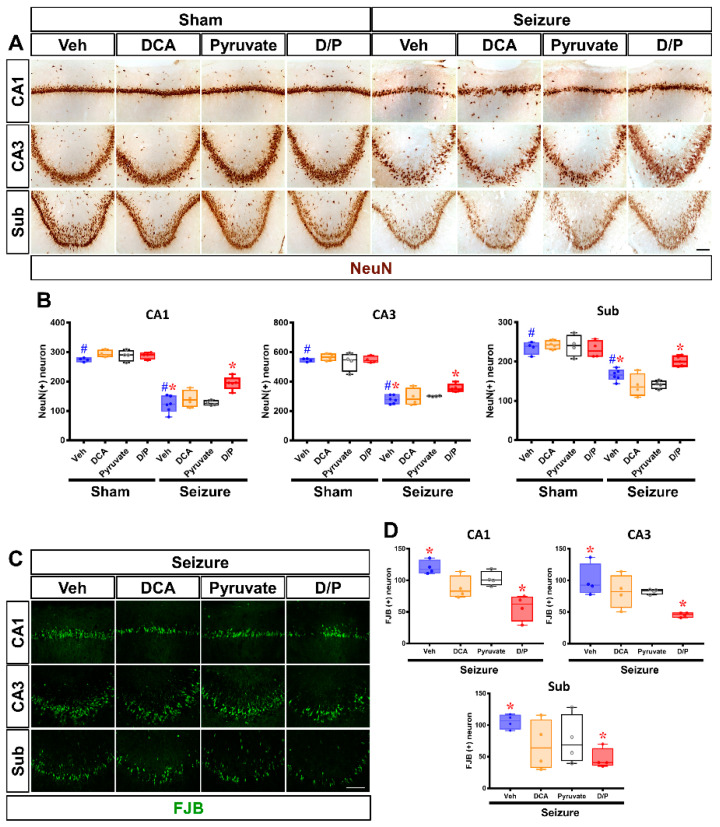
Effects of dichloroacetic acid (DCA) and pyruvate co-treatment on neuronal cell death. DCA and pyruvate co-treatment reduces seizure-induced neuronal cell death. (**A**) DCA and pyruvate co-treatment was evaluated by neuronal nuclei (NeuN) staining to confirm the survival of hippocampal neurons after seizure. There were significant differences between the seizure–vehicle and seizure–DCA + pyruvate treatment groups. DCA and pyruvate co-treatment improved neuronal survival in each area of the hippocampus (CA1, CA3 and Sub) after seizure. Scale bar = 100 μm. (**B**) Bar graphs represent quantified NeuN (+) neurons. y axis: number of surviving neurons (Kruskal–Wallis test followed by a Bonferroni post hoc test: CA1 chi square = 20.332, *df* = 7, *p* = 0.005; CA3 chi square = 19.662, *df* = 7, *p* = 0.006; Sub chi square = 19.494, *df* = 7, *p* = 0.007) blue: vehicle, yellow: dichloroacetic acid (DCA), black: pyruvate, red: DCA and Pyruvate co-treatment. Data are the mean ± S.E.M. *n* = 4 for each sham group. *n* = 5–7 for each seizure group. (**C**) Representative images of degenerating neurons (FJB; green) in the CA1, CA3 and Sub from hippocampus of vehicle and DCA and pyruvate co-treatment groups after seizure. Scale bar = 100 μm. (**D**) Quantification of the number of FJB (+) cells from hippocampal CA1, CA3 and Sub areas. y axis: number of FJB (+) cells (Kruskal–Wallis test followed by a Bonferroni post hoc test: CA1 chi square = 11.515, *df* = 3, *p* = 0.009; CA3 chi square = 9.558, *df* = 3, *p* = 0.023; Sub chi square = 5.779, *df* = 3, *p* = 0.029) blue: vehicle, yellow: dichloroacetic acid (DCA), black: pyruvate, red: DCA and Pyruvate co-treatment. Data are the mean ± Standard Error of the Mean (S.E.M.). *n* = 5–7 for each seizure group. ^#^ Significantly different from the sham vehicle and seizure vehicle-treated group, * Significantly different from the vehicle-treated group; * *p* < 0.05.

Neuronal nuclei (NeuN) staining was performed to determine whether it was possible to increase neuronal cell survival after seizure when dichloroacetic acid (DCA) and pyruvate co-treatment was performed. NeuN is a well-known marker that is exclusively detected in mature neurons, and it is known to generate neuron specific antibodies. We quantified the number of surviving neurons in the hippocampus. Compared with the number of NeuN (+) cells in the sham group, it was confirmed that the number of NeuN (+) cells was significantly reduced in all regions of the hippocampus, cornu ammonis 1 and 3, dentate gyrus, and subiculum (CA1, CA3 and Sub, respectively), in the seizure–vehicle group. After comparing the number of surviving neurons in the seizure–vehicle and DCA and pyruvate co-treatment groups, the number of NeuN (+) cells was found to be significantly higher in the DCA and pyruvate co-treatment group than in the vehicle treatment group (Figure 6A,B. Compared to the seizure–vehicle group, the surviving neurons in the seizure–DCA and pyruvate combination treatment group increased in CA1 by 36.8%, in CA3 by 21.7% and in Sub by 16%). Fluoro-Jade B (FJB) staining was performed to confirm hippocampal neuronal cell death. The number of FJB (+) cells was found to be significantly lower in the seizure DCA and pyruvate co-treatment group than in the seizure vehicle treatment group (Figure 6C,D. Compared to the seizure–vehicle group, the FJB (+) cells in the seizure–DCA and pyruvate combination treatment group decreased in CA1 by 52.7%, in CA3 by 54.4% and in Sub by 63.7%).

### 3.6. DCA and Pyruvate Co-Treatment Inhibitor Reduces Seizure-Induced Cognitive Impairment

**Figure 7:**
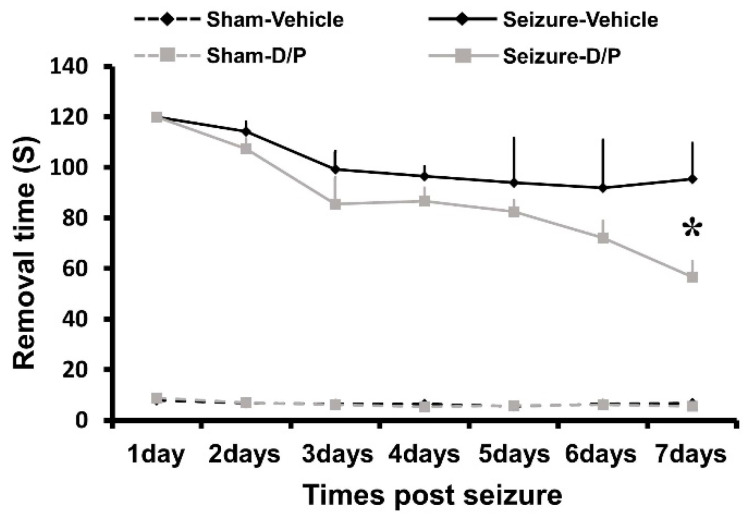
Dichloroacetic acid (DCA) and pyruvate co-treatment cognitive impairment. DCA and pyruvate co-treatment was evaluated using the tape removal test to confirm the changes in cognitive and sensory function after seizure. At day 7 there were significant differences between the seizure–vehicle and seizure–DCA + pyruvate treatment groups. y axis: tape removal time (maximum 120 s) (Kruskal–Wallis test followed by a Bonferroni post hoc test: chi square = 10.484, *df* = 3, *p* = 0.015). Data are the mean ± Standard Error of the Mean (S.E.M.). *n* = 5 for each sham group. *n* = 8 for each seizure group. * represents significant difference between the Seizure Vehicle-treated group and Seizure DP-treated group at day 7; *p* < 0.05.

To investigate the effect of dichloroacetic acid (DCA) and pyruvate co-treatment on seizure-induced sensory and nerve function loss, the experimental animals were behaviorally analyzed using the adhesive removal test method (also known as the tape removal test, TRT). This test was conducted for 1 week from the following day after seizure to the day of sacrifice. Initially, the difference between the seizure–vehicle group and the seizure–DCA and pyruvate co-treatment group was not significant. However, as a result, the seizure–vehicle group had a tape removal time of nearly 100–120 s, and the seizure–DCA and pyruvate co-treatment group removed the tape faster than the seizure–vehicle group. At day 7, TRT time was significantly increased by 88.5 s in the seizure-induced group compared to the sham-vehicle group. However, in the seizure group treated with DCA and pyruvate co-treatment, the TRT time was reduced by 38.7 s compared to that in the seizure–vehicle group (Figure 7).

## 4. Discussion

**Figure 8:**
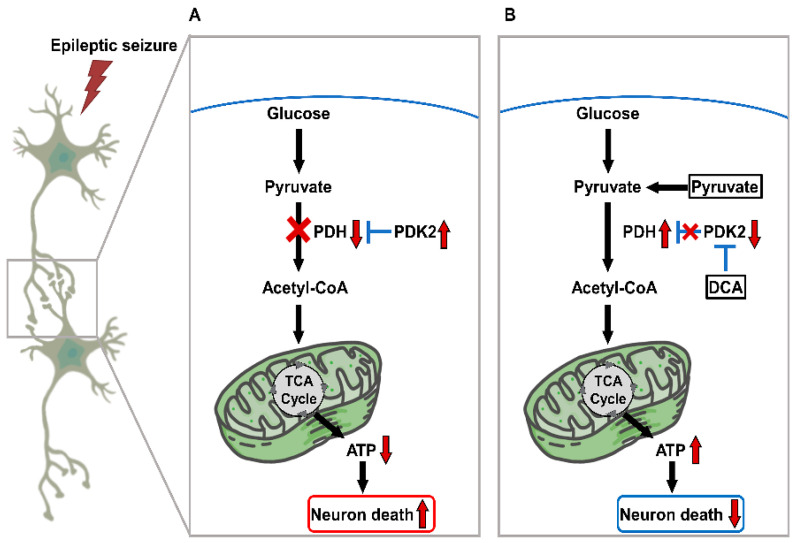
An illustration suggesting the effects of DCA and pyruvate co-treatment on the process of seizure-induced neuronal death. (**A**) After the seizure induced by pilocarpine, the mechanism of neuronal cell death due to blocking of the process at the stage of converting pyruvate into Acetyl-CoA for energy production was examined. (**B**) After seizure, DCA and pyruvate co-treatment inhibited abnormally increased PDK2 activity, increasing the conversion rate of Acetyl-CoA from pyruvate to reduce neuronal cell death.

In this study, we investigated whether a combination treatment of dichloroacetic acid (DCA) and pyruvate would have neuroprotective effects in the hippocampus after seizure. DCA has neurotoxic effects when used at high doses (over 500 mg/kg) or when 300 mg/kg is administered for 10 weeks or longer [42,43]. In previous animal studies, the beneficial effect of DCA administration at a dose of 50–200 mg/kg was confirmed [44,45,46], and this concentration was determined by confirming that treatment at a dose of 100 mg/kg is also beneficial [29,47]. It has been found that low doses of DCA or pyruvate alone do not significantly reduce neuronal cell death due to seizure [48]. However, co-treatment with DCA (a pyruvate dehydrogenase kinase inhibitor) and pyruvate has been found to have neuroprotective effects. These findings suggest that a combination of DCA and pyruvate could be used as post-seizure treatment (Figure 8).

Status epilepticus (SE) is an epileptic seizure that lasts for a long time or is repeated several times. Seizures can cause the widespread necrosis of nerve cells in vulnerable areas of the brain, which has been demonstrated in neuropathology studies on humans and animals [49]. The vulnerable brain regions during a seizure are the hippocampus, amygdala, cerebellar cortex, thalamus, and cerebral cortex. Through the development of medical technology, such as brain wave scanners and neurophysiology, it has been found that seizures caused by epilepsy are due to the temporary and irregular abnormal excitation of neurons. Currently, because of the ability to use drugs to suppress this phenomenon and the possibility of removing the lesions that cause it, this phenomenon is considered a treatable and mitigating disease.

The production of ROS by active oxygen and the production of NADPH oxidase after seizure are important drivers in many neurological diseases, including epilepsy, and are especially important causes of neuronal cell death. When ROS are produced, they directly damage proteins and nucleic acids and activate the poly ADP-ribose polymerization enzyme. In addition, the production of free radicals by epilepsy can cause epileptic seizures. Oxidative stress due to epilepsy mainly occurs in the mitochondria. As epilepsy continues due to mitochondrial oxidative stress and impaired activity, many peroxides are produced, and the antioxidant defenses inside the mitochondria break down. Based on this scenario, our laboratory previously searched for drugs that have good effects, such as neuronal death reduction, inflammatory activation, and neuronal cell survival improvement.

Most of the anti-epileptic drugs are known to work either on sodium and calcium channel currents stabilization (Phenytoin, Valproate, etc.) [50,51] or work to mimic or increase GABA (Gabapentin, Tiagabine, etc.) [52,53]. Moreover, nootropics (Piracetam, Levetracitam, etc.) [54,55,56] and antioxidants are given to some chronic epilepsy patients to take care of neuronal inflammation and oxidative stress. Previous studies have shown that chronic seizure activity reduces adenosine triphosphate (ATP) level in the brain during seizure activity [57,58,59,60,61] and we also confirmed it in this study. Therefore, the clinical significance of the present findings is that co-treatment of DCA and pyruvate may have clinical therapeutic potential by increased brain ATP production, which later provide neuroprotection after the seizure. 

Energy metabolism is specifically impaired in neurons during a seizure [61,62]. Supplementing ATP to improve cell survival appears to be a rational strategy. A ketogenic diet is one of several methods that can be used to increase the level of ATP that has been lowered after a seizure [63,64]; however, this study administered DCA and pyruvate co-treatment to achieve more effective ATP production after seizures. DCA is an effective drug that inhibits pyruvate dehydrogenase kinase activity by activating the pyruvate dehydrogenase complex. DCA is known to increase energy metabolism by inhibiting PDK during this process and is a substance known to increase pyruvate absorption into the mitochondria. Pyruvate is an important compound in chemistry and the last product of the glycolysis process, it is oxidized to carbon dioxide in a series of reactions called TCA circuits, and it is used as the main metabolite of glucose in the process of generating additional energy. Our previous study showed that the administration of pyruvate and DCA also reduces brain damage [23,29,47].

We hypothesized that Pyruvate dehydrogenase kinase (PDK) activation after seizure blocks the entry of pyruvate into the citric acid circuit in mitochondria, reducing ATP formation, leading to neuronal cell death during seizures. During glycolysis, glucose-6-phosphate is converted to ribose-5-phosphate, which can simultaneously produce NADPH (produce superoxide). Therefore, we used pyruvate, not glucose, for the treatment and applied it as energy for ATP production.

Pathological disorders of dysfunction in mitochondrial metabolism have been identified after traumatic brain injury, seizure, ischemia, and hypoglycemia [65,66,67]. It has been reported that determine pyruvate dehydrogenase (PDH) is important for the changes in brain energy metabolism seen in various brain injuries [14,68,69], and DCA, a structural analogue of pyruvate, is attached to the pyruvate binding site to inhibit PDK in the order of PDK2 > PDK1, PDK4 > PDK3 [12]. In this experiment, we confirmed that the level of PDK2 increased in the hippocampus after seizure, and, thus, PDH decreased. However, we showed that DCA and pyruvate co-treatment decreased the PDK2 level in the hippocampus, while the PDH level increased.

Immunohistochemistry and immunofluorescence staining were performed to determine whether the DCA and pyruvate co-treatment would have a neuroprotective effect. When hippocampal neuron survival was evaluated using neuronal nuclei (NeuN) staining in this study, it was found that the administration of only DCA or pyruvate once a day for one week after seizure was ineffective compared to the results of the seizure group. Subsequently, we evaluated the degenerating neurons in the hippocampus by FJB staining. This process confirmed that DCA and pyruvate co-treatment significantly reduced degenerating neurons after seizure. We found that the DCA and pyruvate co-treatment improved the survival of hippocampal neurons as well as decreased hippocampal neuronal death.

After seizure, excessive NADPH oxidase production occurs in the mitochondria, producing excessive reactive oxygen species (ROS). Therefore, in this study, we performed 4-hydroxy-2-nonenal (4HNE) staining to show how DCA and pyruvate reduce oxidative stress [70,71]. In this study, ROS production increased in all regions of the hippocampus after a pilocarpine-induced seizure. These results are consistent with those of several studies that show that seizure induces the excessive production of ROS, causing oxidative stress [71,72]. Through 4HNE staining, we confirmed that the combined administration of DCA and pyruvate reduced oxidative stress. It was hypothesized that this phenomenon would reduce neuronal cell death by reducing seizure-induced ROS.

Brain damage and neurological disorders caused by seizures, traumatic brain injury, ischemia, and hypoglycemia trigger neuro-inflammatory responses and promote metabolic changes, including metabolic shifts [73,74]. Alterations in glycolysis lead to the promotion or inhibition of nerve inflammation [75]. Microglia can be activated immediately when brain damage occurs; the inflammatory cytokines TNF-α and IL-1β are then produced. Activated microglia (including astrocytes) migrate to the damaged area [76,77,78]. Reducing the excessive activation of disease-induced brain inflammation has been considered a potential target for disease treatment. Therefore, when DCA and pyruvate were administered, reduced the activation of microglia and astrocytes, which can exacerbate neuroinflammation and contribute to neurodegeneration after seizure.

## 5. Conclusions

In the present study, supports the hypothesis that the co-treatment of dichloroacetic acid (DCA) and pyruvate has a neuroprotective effect on seizure-induced neuronal death. Therefore, our findings suggest that the co-treatment of DCA and pyruvate may be a clinical significance potential therapeutic approach to neuronal cell death induced by seizure-induced energy metabolism disorders.

## Data Availability

Not applicable.
